# Individual values and well‐being: The moderating role of personality traits

**DOI:** 10.1002/ijop.12751

**Published:** 2021-03-09

**Authors:** Agnieszka Bojanowska, Beata Urbańska

**Affiliations:** ^1^ Department of Psychology SWPS University of Social Sciences and Humanities Warszawa Poland

**Keywords:** Values, Personality, Subjective well‐being, Eudaimonic well‐being

## Abstract

This study examined the role of values, traits and their interactions for the experience of eudaimonic and hedonic well‐being. First wave studies on value and well‐being relationships yielded inconsistent results suggesting that these relationships are moderated by other factors, possibly by personality traits. We asked a representative sample of adult Poles (*N* = 1161) to report on their personality traits (according to five‐factor theory), values (conceptualised by Schwartz) and well‐being (hedonic and eudaimonic). Results showed, that higher Extraversion, Emotional stability, Intellect, Agreeableness and Conscientiousness were related to higher well‐being, confirming and expanding claims from personality theory of subjective well‐being: stable predispositions are related not only to subjective, but also to eudaimonic well‐being. Values expressing Openness to change, Self‐transcendence and Conservation were also positively correlated with well‐being, while the role of Self‐enhancement was unclear. This confirmed that growth needs expressed in Openness to change and Self‐transcendence values promote well‐being, but also that values expressing deficiency needs can be positively related to well‐being, possibly in specific circumstances. Finally, the two levels of personality (traits and values) proved to have a joint relationship to well‐being: higher Conscientiousness and Agreeableness enhanced positive relationships of Openness to change and Self‐transcendence with some aspects of well‐being.

Various aspects of personality may impact well‐being. Certain traits facilitate well‐being and in some cases the data turned out to be so consistent that researchers coined the term “happy personality” to denote configurations of traits that facilitate well‐being (Costa & McCrae, 1980). However, personality does not only consist of traits, but also of aspects related to individual identity that are expressed in the level of values (McAdams & Pals, 2006). Values are also related to well‐being, but not as directly as traits—studies conducted so far are inconclusive as to the role of values for well‐being (Sortheix & Schwartz, 2017). Some value dimensions seem more “healthy,” but these effects do not hold across samples and well‐being conceptualisations (see Bojanowska & Piotrowski, 2018), which suggests that the relationships between values and well‐being may be moderated by other variables. These moderators may be related to people's external circumstances, such as their culture or socio‐economic background (Sortheix & Lönnqvist, 2015) or they may be internal, that is, coming from within the person's personality. This second approach, suggesting that personality traits and values may be analysed jointly in the context of well‐being is especially interesting, given that both values and traits can be framed within the broader and current approaches to personality, such as that proposed by McAdams and Pals (2006). Traits and values come from two distinct theoretical traditions (Nilsson, 2014), but they are related and there is a strong need to integrate these two traditions within common research. For example, Fisher and Boer (2015) showed, that trait Agreeableness is aligned with Self‐transcendence values (e.g., benevolence) and that trait Openness to experience is inversely correlated with Conservation values (e.g., tradition). The question then emerges: What is the relationship of such value‐traits configurations with well‐being? If each of the personality levels has its own specific link to well‐being, does their joint relationship differ in some way or is it merely the sum of its parts? It is possible, that some configurations of values and traits are more compatible with one another and therefore, they may make everyday functioning smoother and easier, which may lead to higher well‐being.

In the present article we analyse values, personality traits and their interactions and consider their role for various aspects of well‐being. As stated above, there are studies on the relationships between values and well‐being (Schwartz & Sortheix, 2018) and on personality traits and well‐being (Steel et al., 2008). A significant proportion of these studies, however, relies on the conceptualisation of well‐being as a compound of satisfaction, positive affect and negative affect labelled subjective well‐being (Diener, 2000), some other studies also include psychological well‐being conceptualised by Carol Ryff (1989; see Anglim et al., 2020 for a meta‐analysis). Little, however, is known about the relationship between traits, values and other conceptualisations of well‐being, such as the unidimensional concept of eudaimonic well‐being, which expresses a person's experience in terms of realising one's potential (Waterman et al., 2010). Secondly, although there is data linking traits and values (Fisher & Boer, 2015), little is known about their joint significance for well‐being. In the present study, we aim to fill these two gaps by including values and traits simultaneously, and by analysing their interactions for subjective and eudaimonic well‐being.

## Subjective and eudaimonic well‐being

Questions about the conceptualisation of human happiness have been present in psychology for a long time. It is difficult to find one answer to what happiness is. In the present article we narrow this down to two conceptions of well‐being: subjective (hedonic) and eudaimonic. They both express the individual experience of well‐being but differ quite substantially in their theoretical underpinnings. In the hedonic or subjective well‐being conception (Diener & Ryan, 2009), people evaluate their lives according to their own criteria and report on their satisfaction with life. The advantage of this approach is that each person can place more or less weight on determinants of their satisfaction consistently with their preferences and values. Reporting satisfaction is therefore considered a cognitive process that needs to be accompanied by an emotional one—subjective well‐being also includes positive and negative affective states experienced in everyday life.

In contrast, eudaimonic well‐being is defined as living well or actualising one's potential (Deci & Ryan, 2006). From this perspective well‐being is not a subjective state, but a process of fulfilling a person's “daimon” or true nature. Waterman et al. (2010) describe aspects of eudaimonic well‐being such as self‐discovery, a sense of purpose and meaning in life, intense involvement in activities and investment of significant effort.

## Personality traits and well‐being

According to five‐factor theory (McCrae & Costa, 2008), basic personality traits express people's basic tendencies and traits can be inferred indirectly from behaviour (or self‐reports). Traits determine individual choices, preferences and behaviours (McCrae & Costa, 1991). For example, extroverted and emotionally stable people are more often involved in social or pleasure‐related behaviours; they find it easier to engage in new relationships, which in turn affects their emotions and satisfaction. Some traits are directly related to emotional functioning and therefore may impact affective states (e.g., Emotional stability), some may determine how easy it is for a person to realise various goals that may promote satisfaction (e.g., Conscientiousness), others facilitate seeking new activities and context that may help realise one's potential (e.g., Openness to experience).

Studies show that the five traits are one of the most consistent predictors of well‐being (Lucas, 2018), although recent reports also suggest that the direction of influence may be inverse (Soto, 2015). Nevertheless, higher level of subjective well‐being is associated with higher Emotional stability, Openness to experience, Extraversion, Conscientiousness and Agreeableness and they explain up to 46% of variance in different well‐being aspects (Anglim et al., 2020; Fetvadjiev & He, 2019). Our first hypothesis is therefore consistent with the above data: higher Emotional stability, Openness to experience, Extraversion, Conscientiousness and Agreeableness will be positively related to well‐being (H1).

## Basic human values and well‐being

Personality is not limited only to traits. Human identity is also based on what people believe to be important and worth pursuing, and this is best expressed by individual value hierarchies. One of the most renowned conceptions of human values was developed by Schwartz, who defines them as “trans‐situational goals, varying in importance, that serve as guiding principles in the life of a person or group” (Schwartz et al., 2012, p. 3). They guide human behaviour and serve as standards or criteria of what is good or bad, worth doing or avoiding—depending on which values are important for a person (Schwartz, 2012).

Schwartz identified a catalogue of values that are universal and basic (Schwartz, 2007) and this catalogue forms a circular motivational continuum. Adjacent values can be pursued simultaneously, because they share motivational meanings. The most recent set of basic values confirmed in intercultural research includes 19 values (Schwartz et al., 2012), that can be grouped into four dimensions: Openness to change, Self‐enhancement, Conservation and Self‐transcendence. The grouping into higher order dimensions is an expression of the patterns of conflict and congruity between values—values that lie close to one another express similar core motivations and therefore can be grouped together. Values that belong to the same higher order dimension can also be realised simultaneously, for example, in one activity. Values that lie on opposite sides of the circle contradict one another (e.g., Openness to change contradicts Conservation; Schwartz, 2012).

People who value Openness to change find it important to be self‐directed in thought and action, and to seek out pleasure and stimulation in their lives. Those who value Self‐enhancement tend to strive for achievement and power over other people or resources. Those for whom Conservation is important value humility, security and tradition, and they tend to follow rules and conform to other people. Finally, people who value Self‐transcendence would say that one should be tolerant, care for nature and society and act with benevolence towards other people. With these descriptions in mind it seems natural, that some values stand in opposition, while others may be realised at the same time. A person for whom seeking stimulation is important would probably place little value in security. The table below shows the sets of values that constitute the four higher order dimensions.

Sortheix and Schwartz (2017) described mechanisms by which values can be associated with subjective well‐being. They stated that achieving healthy values can lead to assessments, attitudes and behaviours that promote well‐being. For example, people for whom benevolence is important think that people are nice, they tend to be tolerant of others and committed to helping them and these convictions and behaviours would lead to an enhanced well‐being. Studies conducted since the 1990s, although there are not many of those, showed that some values are indeed a little “healthier.”

It seems that Openness to change may enhance well‐being, because values of self‐direction constituting this dimension express growth orientation that motivates people to engage in activities related to self‐actualisation, expression of ideas, abilities and feelings and to satisfy the need for autonomy (Schwartz & Sortheix, 2018). Those values should therefore be positively related to well‐being, especially to its eudaimonic aspect (see Bojanowska & Piotrowski, 2018). Additionally, values of stimulation and hedonism could be related to subjective well‐being, especially to the pleasant affect because they motivate people to seek out new and pleasant experiences.

Relationships between Self‐enhancement and well‐being are unclear. Some researchers (Schwartz & Sortheix, 2018) claim that Self‐enhancement may facilitate well‐being because it expresses a personal focus (as opposed to focusing on others), which includes valuing achievement and power, so people motivated by these values may be driven towards goal realisation. Achieving their goals may lead to enhanced satisfaction. On the other hand, valuing achievement and power may inhibit the ability to maintain positive relationships with other people. A person for whom domination is a key element in their value hierarchy may be difficult in relationships, as cooperation would not be their default stance and they would rather focus on competition. This was confirmed in one study with reference to eudaimonic well‐being (Bojanowska & Piotrowski, 2018).

The function of Conservation values for well‐being is also unclear. Sortheix and Schwartz (2017) suggest, that it expresses a self‐protection orientation (as opposed to growth) and therefore it may not be beneficial for well‐being, because it reflects the need to avoid danger and anxiety and motivates to submit to the expectations of society or to ensure control and dominance to overcome fear. It also expresses a focus on others (as opposed to personal focus) and therefore it directs attention to social requirements and obligations that limit autonomy.

Similar statements with regard to focus on others can be formulated in reference to Self‐transcendence, which expresses values of benevolence and universalism. However, studies tend to show (Bojanowska & Piotrowski, 2018; Cohen & Shamai, 2009; Sortheix & Schwartz, 2017) that Self‐transcendence is positively related to well‐being, mostly through positive social relationships.

These studies are not conclusive and other reports yielded contradictory results (e.g., Buchanan & Bardi, 2015), so we do not formulate a specific hypothesis, rather, we pose a question on how the dimensions of values are related to different well‐being dimensions (Q1). The inconsistencies found in the mentioned reports led to a conclusion that moderating factors influence relationships between values and well‐being. They may refer to the level of economic development in the country where the data was collected (Sortheix & Schwartz, 2017), person‐environment congruence in values (Sortheix & Lönnqvist, 2015), temperament traits (Bojanowska & Piotrowski, 2018) or personality (Haslam et al., 2009). In the present article we focus on the possible moderating role of personality traits.

## Personality, values and well‐being

Personality traits and values are distinct but related to each other (McAdams & Pals, 2006; Roccas et al., 2002): traits refer to what people are like and how they usually behave, while values refer to what is important for people and to their goals. Although traits and values may share similar biological foundations and until recently there was an overemphasis placed on the socialisation processes in the formation of individual values, these two levels of personality remain to be distinguishable from one another. They are either distinguished by the proportion of genetic factors that shape them (McAdams & Pals, 2006) or by the processes that govern them, that is, while values seem to express conscious strivings, traits express both conscious and unconscious motivations (Fisher & Boer, 2015).

Consequently, traits and values can be considered related but distinct. Some personality traits share similar motivations with some of the values and we argue that consistency between traits and values that overlap conceptually might produce higher well‐being, because people are then motivated to engage in certain activities by their values and these activities are consistent with their preferences stemming from their traits. As indicated by Fisher and Boer (2015), Openness to experience is positively associated with Openness to change and Self‐transcendence values, while Extraversion is related positively with Openness to change and Self‐enhancement and these relationships are explained by the approach tendencies that underly both these traits and that can be satisfied through seeking novelty (Openness to change), aiming for achievements (Self‐enhancement) or willingness to engage in positive relationships with other people (Self‐transcendence). They also showed that Agreeableness correlates positively with Self‐transcendence values and negatively with Self‐enhancement, which seems to express a tendency to engage in cooperation rather than competition and this can be achieved by realising goals attached to Self‐transcendence values (e.g., benevolence, universalism). Finally, they showed that Conscientiousness is related positively with Conservation values, negatively with Openness to change values, and this can be explained by the underlying impulse control or dutifulness expressed in trait Conscientiousness and consistent with goals attached to Conservation (e.g., observing tradition). Neuroticism was found to be only marginally related to values.

We expect to find similar correlations in our sample (H2). Further, we expect consistency between traits and values to be associated with higher well‐being (H3). Building upon the goal‐based approach to personality (Mischel & Shoda, 1995), traits and values determine the goals that a person sets. If these goals simultaneously fit in well with traits and values, their realisation may be easier (e.g., higher levels of well‐being would be observed when an extrovert values Openness to change or Self‐enhancement or when a highly conscientious person values Conservation, compared to other configurations).

As pointed by Sortheix and Lönnqvist (2015), the correlations between values and well‐being may be moderated by social contexts and intercultural differences—they may be different in highly developed countries, where cooperation brings forth well‐being more as opposed to underdeveloped countries where people have to compete for resources. This context is not only crucial to the relationships between values and well‐being, but also for the relationships between values and personality (Fisher & Boer, 2015). Although this is not an intercultural study, the socio‐cultural context of the sample is important for framing the results within the current literature on the subject.

The present study has significant implications for theory. Firstly, relationships between personality and subjective well‐being would further confirm the personality theory of subjective well‐being (Costa & McCrae, 1980), whereas relationships between personality and eudaimonic well‐being would suggest that this theory can be expanded to also engulf other well‐being expressions. Consequently, the functions of personality traits could turn out to also regulate behaviours leading to the realisation of one's potential, possibly through the engagement in specific behaviours.

Secondly, relationships between values and well‐being would confirm that the cognitive (attitudes towards the world and other people) and behavioural (what activities people engage in) underpinnings of values can be favourable or unfavourable. The patterns of these relationships will also have consequences for the understanding of the underlying mechanisms that determine which values people deem important: positive relationships between Self‐transcendence or Openness to change to well‐being would confirm that expressions of growth needs (Sortheix & Schwartz, 2017) are more favourable to well‐being. This is especially interesting for eudaimonic well‐being, that has realisation of one's potential (Waterman et al., 2010) at its core—a dimension that may require a growth stance.

Finally, if interactions between values and personality are indeed related to well‐being, this would mean that the regulatory functions of traits that express a more basic level of personality (traits; more closely related to human biology; McAdams & Pals, 2006) can support or hamper strivings related to its higher levels (values). In other words, traits regulate “how” people engage in behaviours (e.g., conscientiously, with little anxiety), whereas values regulate “what” they engage in and these two levels are intertwined. This would further elucidate the relationships between the various levels of personality.

## METHOD

### Participants and procedure

The final sample consisted of *N* = 1161 Polish adults (55% women) aged between 18 and 78 years (*M* = 45, *SD* = 15.01). The study was conducted online via a professional research panel. The participants signed up with the panel for various studies. They get points for participation, which they can later exchange for small “gifts” (household appliances, etc.). The data were collected from a national sample representative of gender, age, education and the regions of Poland. We included two control questions (asking participants to indicate a specific answer, e.g., “4”) and excluded data with extremely short response times or no variation between answers. In this way, we removed 15% of the initial data. After examining the demographics of the removed participants, we then collected additional data to compensate for these (the *N* = 1161 is the final sample). Each participant provided written informed consent. The study was approved by an Ethical Committee. All procedures performed in studies involving human participants were in accordance with the ethical standards of the institutional research committee and with the 1964 Helsinki Declaration and its later amendments or comparable ethical standards. Informed consent was obtained from all individual adult participants included in the study.

### Measures

*Questionnaire of Eudaimonic Well‐being* measures eudaimonic well‐being (Waterman et al., 2010, adapted by Kłym‐Guba & Karaś, 2018). It contains of 21 items (e.g., “I believe I have discovered who I really am”) with a 7‐point scale, from 1—*I definitely disagree* to 7—*I definitely agree*. High scores express high eudaimonic well‐being. This scale had satisfactory reliability, with Cronbach's *α* = .88.

*Satisfaction with life scale* is a five‐item scale created to measure life satisfaction (Diener et al., 1985). Participants indicate how much they agree or disagree with each item (e.g., “In most ways my life is close to my ideal.”) on a scale from 1—*strongly disagree* to 7—*strongly agree*. High scores express high satisfaction. This scale had satisfactory reliability, with Cronbach's α = .90.

*The Positive and Negative Affect Scale* (Watson et al., 1988) measures positive and negative affect. The scale consists of 10 adjectives reflecting positive affect (e.g., enthusiastic, excited) and 10 reflecting negative affect (e.g., upset, scared). Respondents are asked to indicate the extent they had felt this way over the past 2 weeks from 1 (*only slightly or not at all*) to 5 (*extremely*). The scales showed satisfactory reliability, with Cronbach's *α* = .86 for positive affect and *α* = .91 for negative affect.

To assess basic human values according to Schwartz's model with 19 values, we used the revised Portrait Values Questionnaire, adapted to Polish by Cieciuch (2013). It consists of 57 items—three items per value. Values were then grouped into four dimensions (see Table 1): Openness to change, Self‐enhancement, Conservation and Self‐transcendence. Respondents assessed how similar they are to the person described in each item (e.g., “Doing everything independently is important to him.”). Answers are given on a 6‐point scale from 1 (*not like me at all*) to 6 (*very much like me*). Reliability was satisfactory for all four scales: Self‐enhancement, α = .86; Openness to change, α = .85; Conservation, α = .90; and, Self‐transcendence, α = .91.

**TABLE 1:** The four higher order dimensions of values

Dimension	Values
Openness to change	Self‐direction thought, self‐direction action, stimulation, Hedonism^a^
Self‐enhancement	Achievement, power‐dominance, power‐resources
Conservation	Humility, conformity‐interpersonal, conformity‐rules, tradition, security‐personal, security‐societal, face
Self‐transcendence	Universalism‐tolerance, universalism‐nature, universalism‐concern, benevolence‐caring, benevolence‐dependability

^a^ Hedonism can either be included in Openness to change or self‐enhancement. In this study it is included in Openness to change, because it correlates values from this group more strongly than to values from self‐enhancement.

IPIP‐BMF‐20 (*The International Personality Item Pool*, Donnellan et al., 2006) was used to assess personality traits (Extraversion, Agreeableness, Conscientiousness, Emotional stability and Intellect; adapted by Topolewska et al., 2014). There are 20 statements and respondents rate each statement on a scale from 1—*very inaccurate* to 5—*very accurate*, indicating if the statement describes their personality. Reliability was satisfactory for all scales: Extraversion α = .80; Agreeableness α = 0.70; Conscientiousness α = .68; Emotional stability α = 0.73; and Intellect α = 0.67.

## RESULTS

### Descriptive statistics

First, we tested all items for common method bias using Harman's single factor test. The index was 17.1%, which shows that there is no issue with common method bias. Basic statistics and correlations between variables are presented in Table 2. There were significant correlations between almost all traits, value dimensions and well‐being components.

**TABLE 2:** Correlations between values, traits and well‐being

	M	SD	Eud	Sat	PA	NA	ENH	OPE	CONS	TRA	Extrav	Agree	Const	Stab	Intel
Eud	96.7	15.1	1	.36**	.42**	−.41**	−.11**	.41**	.37**	.49**	.36**	.50**	.41**	.33**	.52**
Sat	19.2	5.7		1	.52**	−.36**	.09**	.16**	.11**	.09**	.24**	.11**	.10**	.42**	.19**
PA	31	6.3			1	−.18**	.2**	.35**	.22**	.24**	.29**	.21**	.15**	.31**	.32**
NA	28	7.9				1	.18**	−.15**	−.09**	−.18**	−.30**	−.22**	−.21**	−.57**	−.31**
ENH	3.4	0.85					1	.36**	.14**	−0.04	.09**	−.23**	−.11**	−0.06	−0.04
OPE	4.4	0.63						1	.47**	.59**	.21**	.22**	.17**	.12**	.34**
CONS	4.4	0.67							1	.71**	.07*	.33**	.31**	0.01	.06*
TRA	4.7	0.68								1	.16**	.54**	.28**	.06*	.26**
Extrav	12	3.36									1	.34**	.12**	.36**	.37**
Agree	14.6	2.62										1	.34**	.10**	.33**
Const	14.3	2.82											1	.09**	.24**
Stab	11.5	2.99												1	.32**
Intel	14	2.66													1

*Notes:* Agree = Agreeableness; CON = Conservation value; Const = Conscientiousness; ENH = Self‐enhancement value; Eud = eudaimonic well‐being; Extrav = Extraversion; Intel = Intellect; NA = negative affect; OPE = Openness to change value; PA = positive affect; Sat = satisfaction with life; Stab = Emotional stability; TRA = Self‐transcendence value.

* *p* < .05.

** *p* < .01.

Consistently with Hypothesis 1, higher Emotional stability, Openness to experience, Extraversion, Conscientiousness and Agreeableness were positively related to more favourable indices of all well‐being dimensions. Regarding Question 1 on the relationship between values and well‐being, we found that Openness to change, Conservation and Self‐transcendence were related positively to well‐being, while Self‐enhancement was related to higher satisfaction and negative affect but also to lower eudaimonic well‐being and higher positive affect. Hypothesis 2 was also mostly confirmed: relationships between values and traits in our sample were mostly consistent with findings summed by Fisher and Boer (2015). Agreeableness was positively related to Self‐transcendence and negatively with Self‐enhancement, but in our sample it was also positively related to Openness to change and Conservation. Intellect was positively related to Openness to change and Self‐transcendence. Conscientiousness was related positively to Openness to change (this is inconsistent with earlier findings), Conservation and Self‐transcendence and negatively to Self‐enhancement. Extraversion was positively related to Openness to change and Self‐transcendence (marginally with other dimensions). This last effect is inconsistent with the findings reported by Fisher and Boer (2015) and it shows that in this sample extroverts may tend to set pro‐social goals attached to Self‐transcendence values more often than introverts.

### Traits, values and their interaction—Effects on well‐being

**Figure 1:**
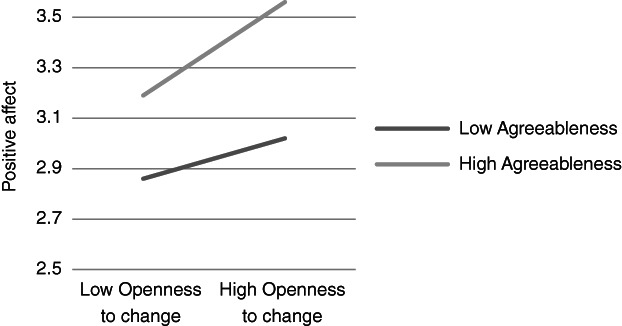
Model 1: Interaction between Agreeableness and Openness to change for positive affect.

**Figure 2:**
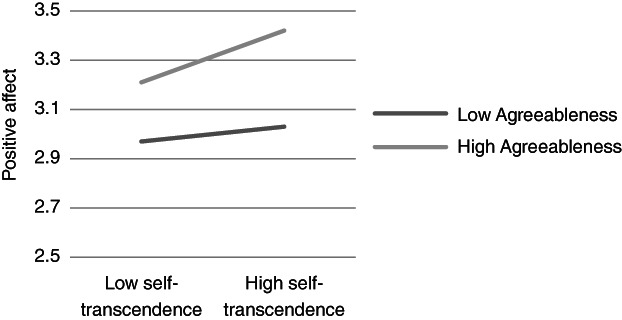
Model 2: Interaction between Agreeableness and Self‐transcendence for positive affect.

**Figure 3:**
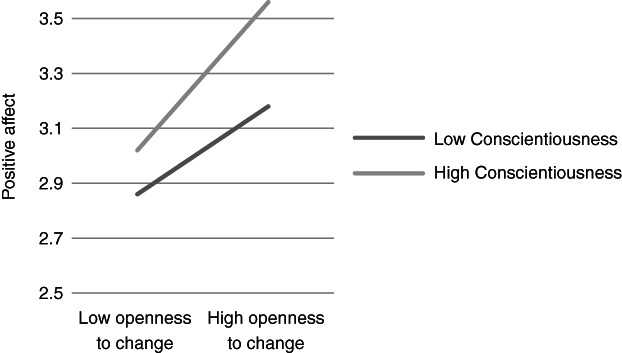
Model 3: Interaction between Conscientiousness and Openness to change for positive affect.

**Figure 4:**
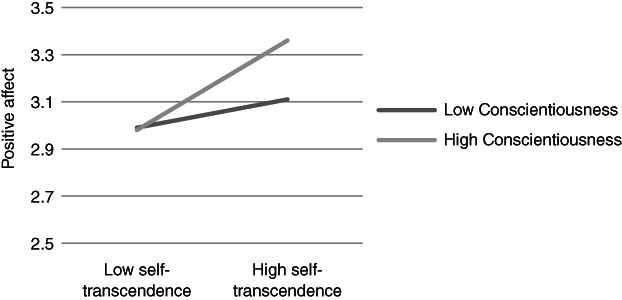
Model 4: Interaction between Conscientiousness and Self‐transcendence for positive affect.

**Figure 5:**
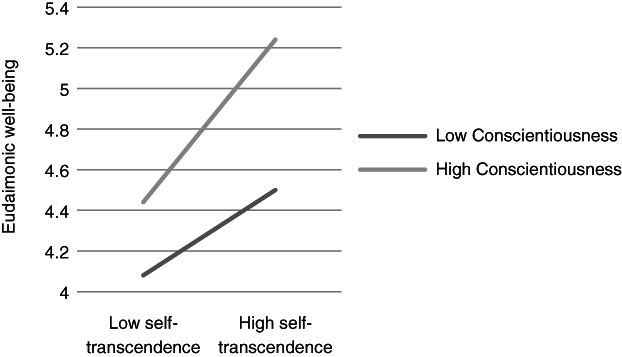
Model 5: Interaction between Conscientiousness and Self‐transcendence for eudaimonic well‐being.

**Figure 6:**
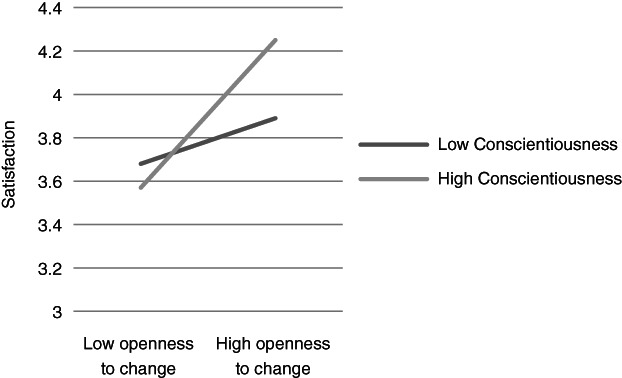
Model 6: Interaction between Conscientiousness and Openness to change for satisfaction.

**Figure 7:**
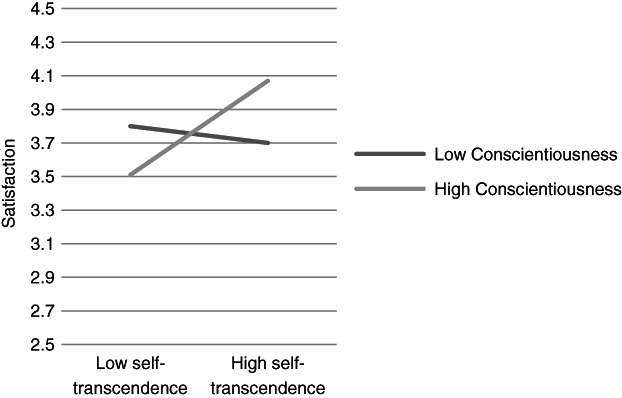
Model 7: Interaction between Conscientiousness and Self‐transcendence for satisfaction.

**Figure 8:**
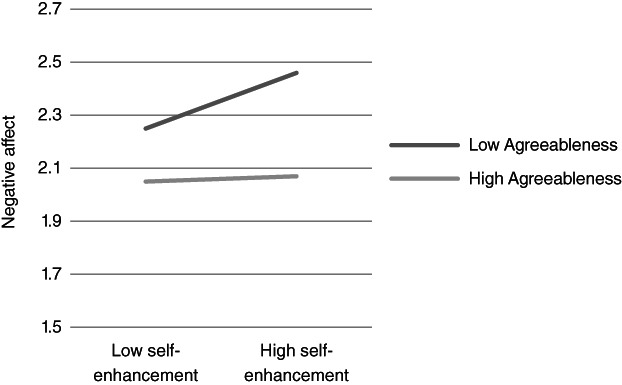
Model 8: Interaction between Agreeableness and Self‐enhancement for negative affect.

We conducted a series of regression analyses using Hayes PROCCESS macro (Model 1, simple moderation)—separately for each well‐being component, and the predictors were entered in sets of two (one value dimension + one personality trait). Because there were a lot of models tested (80 models), we calculated false discovery rate correction to exclude natural fluctuations of data patterns (Benjamini & Hochberg, 1995). This resulted in eight models where the interaction remained significant. For clarity, we only present those eight models: Table 3. presents the effects of traits, value dimensions and their interactions on well‐being. The effects of traits and values are consistent with the pattern found in the correlation analyses. The interactions refer to Conscientiousness (five effects) and Agreeableness (three effects). These interactions are presented in Figures 1, 2, 3, 4, 5, 6, 7, 8.

**TABLE 3:** Regression models: Effects of values, traits and their interaction on well‐being dimensions

	Value (*β*)	Trait (*β*)	Interaction (*β*)	R^2^
Model 1—DV: Positive affect	Openness to change	Agreeableness	Interaction	
	.32**	.12**	.08**	.15**
Model 2—DV: Positive affect	Self‐transcendence	Agreeableness	Interaction	
	.18**	.11**	.08**	.07**
Model 3—DV: Positive affect	Openness to change	Conscientiousness	Interaction	
	.34**	.08**	.10**	.14**
Model 4—DV: Positive affect	Self‐transcendence	Conscientiousness	Interaction	
	.21**	.09**	.08**	.07**
Model 5—DV: Eudaimonic well‐being	Self‐transcendence	Conscientiousness	Interaction	
	.41**	.29**	.07**	.33**
Model 6—DV: Satisfaction with life	Openness to change	Conscientiousness	Interaction	
	.15**	.07*	.08**	.04**
Model 7—DV: Satisfaction with life	Self‐transcendence	Conscientiousness	Interaction	
	.07*	.08**	.09**	.02**
Model 8—DV: Negative affect	Self‐enhancement	Agreeableness	Interaction	
	.13**	−.19**	−.09**	.08**

*Note:* DV = dependent variable. *R* ^2^ = variance of the DV explained by the model.

* *p* < .05.

** *p* < .01. *p*‐values corrected for false discovery rate.

In Models 1, 2 and 8 *Agreeableness* interacted with value dimensions. The shape of these interactions (Figures 1, 2, 8) shows that the most favourable well‐being indices are found when high Agreeableness is accompanied by valuing Openness to change and Self‐transcendence or when low Agreeableness is accompanied by high value placed in Self‐enhancement. In Models 3–7 *Conscientiousness* interacted with Openness to change and Self‐transcendence and the pattern (Figures 3, 4, 5, 6, 7) was consistent: high Conscientiousness accompanied by high Openness to change or Self‐transcendence values was associated with higher well‐being.

## DISCUSSION

We aimed to examine how values, personality traits and their interaction are related to different aspects of well‐being. The data are consistent with earlier findings in terms of the direct roles of values and traits, but it adds new information about their nuanced role for specific well‐being components and about the interactions between values and traits.

### Traits and well‐being

Consistently with earlier findings (e.g., Anglim et al., 2020), higher Agreeableness, Conscientiousness, Intellect, Emotional stability and Extraversion were related to higher well‐being in all of its aspects. Agreeableness, Conscientiousness and Intellect had stronger relationships with eudaimonic well‐being, than with elements of subjective well‐being. In earlier studies, when only the subjective well‐being was taken into account, these three traits seemed to be considered less important for well‐being than Extraversion and Emotional stability. It seems that we managed to find a new mechanism—they are related to well‐being, but their contribution is of a different quality—they contribute to people's sense of purpose and meaning in life, intense involvement in activities, investment of significant effort and self‐discovery (Waterman et al., 2010) more than to satisfaction or pleasurable affect (Diener, 2000). Being agreeable, conscientious and open to experience supports the process of realising one's potential most of all, and to a lesser degree it also contributes to a sense that life brings satisfaction and pleasure. Extraversion had a similar function for eudaimonic well‐being (it also supported it) but it was related to positive affect to a similar degree, which is consistent with the definition and function of this trait (Costa & McCrae, 1980; McCrae & Costa Jr., 2008)—it refers to positive affectivity (therefore it is correlated with positive affect). Emotional stability was the only trait that had strongest correlations with the negative affect and this is consistent with the definition and function of this trait and its biological underpinnings (Costa & McCrae, 1980; McCrae & Costa Jr., 2008).

The above relationships confirm and expand the personality theory of subjective well‐being (Costa & McCrae, 1980). They fully confirm the patterns of these relationships discussed in this theory, that is, that subjective well‐being is mostly linked to Extraversion and Emotional stability and that this has significant implications for stable rank‐order individual differences in well‐being. They also show that the remaining three traits can gain significance in this theory if its conceptualisation of well‐being is expanded to also engulf eudaimonia. This could possibly lead to a new version of this theory, that can be labelled the five trait theory of subjective and eudaimonic well‐being.

### Values and well‐being

Most associations between values and well‐being were significant, but the stronger associations related to eudaimonic well‐being. *Openness to change* was positively related to well‐being, which is consistent with earlier research results and theoretical assumptions (Bojanowska & Piotrowski, 2018; Sortheix & Schwartz, 2017): self‐direction may help people engage in those activities that suit their preferences and a tendency to seek out pleasure and stimulation provide opportunities for a diverse and challenging experience.

*Self‐transcendence* was also positively related to well‐being. This result is inconsistent with assumptions made by Sortheix and Schwartz (2017), who claimed that since Self‐transcendence is a dimension that contributes to the concentration on others (and not on self) and is therefore unbeneficial for well‐being. Our data shows, that it is beneficial—people who value benevolence and universalism have a default cooperative stance, therefore their social relationships may be more rewarding and the quality of relationships is one of the most important elements by which people evaluate their well‐being (Bojanowska & Zalewska, 2016).

*Conservation* was also positively related to well‐being. In numerous studies Conservation was negatively correlated with well‐being, but as pointed out by Schwartz and Sortheix (2018), the relationship between values and well‐being is moderated by person‐environment fit. Poland is a country, in which conservative values are accepted by a large part of the population (Hofstede Insights, 2018) and therefore they can support well‐being (especially eudaimonic). It seems that this context may reward those who are motivated to behave with humility and conformity, engage in behaviours related to traditions and who value security. This is very much consistent with the mainstream public discourse currently observed in Poland and reflected in, for example, the success of conservative political parties.

*Self‐enhancement* values, also related negatively to well‐being in many other studies, turned out to be related with lower eudaimonic well‐being and higher negative affect, but positively related to satisfaction (marginally) with life and positive affect. Self‐enhancement expresses personal focus and self‐protection. Earlier works (Schwartz & Sortheix, 2018) suggested that values falling into the personal focus dimension may have positive impacts, while self‐protection values may have negative impacts on well‐being. This explains why Self‐enhancement is positively related with some aspects of well‐being and negatively with others.

These patterns partly confirm the theoretical assumptions about the underpinnings of values related to growth and deficiency needs: values that express growth needs (*Openness to change* and *Self‐transcendence*; Sortheix & Schwartz, 2017 *)* are positively related to well‐being. Possibly, these values promote well‐being and are their antecedent, but it is also possible that growth needs and higher well‐being both stem from higher availability of psychosocial resources (Hobfoll, 1989), that is, that people who are “better off” are more eager to value growth needs because they do not experience as many deficiencies and that they find it easier to engage in pleasurable or self‐fulfilling activities and this results in higher well‐being. The mechanisms governing the links between deficiency needs and well‐being seem more complex and possibly more sensitive to moderating factors.

### Interactions between traits and values

Some personality traits moderate the relationship between different aspects of well‐being and some of the values. Although the effects found were weak, they were consistent with theoretical assumptions presented in the introduction and with the functions of traits for engagement in various behaviours.

People engage in activities that they deem worth their efforts. Which activities are viewed this way depends on individual values. One person may find meaning in engagement in new and stimulating experiences, while another person would rather devote their time to activities that result in a greater sense of security for them and their loved ones. The traits that those people have may either help them realise their goals smoothly and therefore lead to a greater well‐being or they may become barriers in goal realisation. In our study, we found interesting patterns of these interactions for two traits: Agreeableness and Conscientiousness. Interestingly, these two traits were usually only marginally related to well‐being, but as suggested by McCrae and Costa Jr. (1991), they serve an important function for well‐being: high Agreeableness fosters loving relationships, while high Conscientiousness promotes goal attainment and accomplishments. We found this to be true especially when high levels of these two traits were accompanied by high value placed in Self‐transcendence and Openness to change. This effect is consistent with the main function of these traits for well‐being—higher Agreeableness and Conscientiousness are in themselves beneficial for well‐being (see introduction), but they also seem to serve an additional function for people who scored higher in these values. This means that when people are driven to realise specific goals (such as seeking pleasure or engaging in positive relationships) being more agreeable and conscientious may help them achieve this and through that lead to greater well‐being. This may refer both to the effect of their activities and to the process itself—for example, a person who is more agreeable would probably find ways to strive for what they want in a way that does not cause conflict with other people. As stated by McCrae and Costa Jr. (1991), these two dimensions reflect the Freudian *Arbeit und Liebe*. Being agreeable while valuing benevolence, caring and seeking novelty may help foster positive relationships and lead to greater well‐being (*Liebe*). Being conscientious while valuing these things may foster effectiveness in the attainment of goals attached to these values (*Arbeit*). These effects show that Agreeableness and Conscientiousness might have seemed marginal for well‐being, because the function of these traits manifests itself more strongly when people engage in realising specific values. These results also confirm, that the two levels of personality included in McAdams and Pals's (2006) model are connected and that personality is a system simultaneously determining what people do and how they do it. The “what” is related to values and then realised in a way (the “how”) dictated by traits and that in some cases, this interaction between the content and the form of behaviour is related to well‐being.

## CONCLUSION

This study has three significant contributions to the ongoing debate on personality and well‐being. Firstly, it shows that the well‐established concept of “happy personality” can be expanded further (Costa & McCrae, 1980). If it is broadened to also include eudaimonic well‐being (and not only subjective), it can logically include all five personality traits. Such new, broader direction of research could explain the relationships between traits and well‐being more fully and elucidate the functions of traits for the aspects of well‐being that are related to meaning making and realising one's potential (Waterman et al., 2010).

Secondly, this study furthers the claims stated in the Theory of Basic Human Values (Schwartz, 1992), that values are related to well‐being. The ongoing debate on which values are healthy and which are not has some unclear points. Results presented here are consistent with the hypothesis that values that help foster good relationships (high Self‐transcendence and consequently—low Self‐enhancement; Sortheix & Schwartz, 2017) promote well‐being, and it undermines the hypothesis that values falling into the social focus dimension (as opposed to personal focus) are supposed to be unhealthy (Schwartz & Sortheix, 2018). It also supports the claims that person‐environment fit in terms of values is favourable for well‐being (Sortheix & Lönnqvist, 2015) by showing positive functions of Conservation in the Polish (conservative) context and it confirms the generally healthy role Openness to change values (Schwartz & Sortheix, 2018).

Finally, the results elucidate the hypothesised relationships between lower (traits) and higher (values) levels of personality (McAdams & Pals, 2006) and their joint significance for well‐being. They do not support the consistency hypothesis, that is, that traits and values consistent with one another in terms of their “content” produce higher well‐being (e.g., high Openness to change values with high Openness to experience trait, Fisher & Boer, 2015), rather they show a new direction of research on the functions of Agreeableness and Conscientiousness in the attainment of personally valuable goals.

### Limitations and future agenda

The study has a number of limitations. First, this is a correlational study. We assumed that the directions of relationships go from personality to well‐being, mostly because this is more common in research on personality and researchers tend to interpret data this way, with obvious reservations and an awareness that the direction can be inversed (see e.g., Soto, 2015). Secondly, the interactions we found are weak and they show that these possible moderation mechanisms are subtle and may be limited in scope. Nevertheless they are internally consistent enough and consistent with the functions of traits to be considered plausible. The effect sizes vary, with the strongest effect found in the model that includes eudaimonic well‐being, and the smallest for satisfaction with life. This is consistent with the fact, that satisfaction with life is more strongly correlated with other traits, not included in the presented interaction models (Neuroticism and Extraversion; Costa & McCrae, 1980). To deal with this issue, future studies might aim to identify subsamples where these effects are stronger or look for other moderators of these relationships. One such area of research that needs to be explored further is the study of behaviours that express values and their relationship to well‐being. In this study we only considered what people deem important, but we did not analyse how people realised their values in everyday life. We hypothesise, that values play a more significant role when they are accompanied by behaviours that express them. This is consistent with the core function of values (i.e., determining what is worth pursuing; Schwartz, 2012).

We also conducted this research only in one country (Poland), so possible generalisations are limited. Intercultural studies are needed to discern between effects that are limited to specific socio‐cultural context and those that are universal. This is especially significant since the discussion on the importance of values for well‐being suggests that threat level (Fisher & Boer, 2015), economic development or person‐environment fit in terms of values may play a role (Sortheix & Lönnqvist, 2015).
